# Reply to Meshnick and Hastings et al

**DOI:** 10.1093/cid/ciw584

**Published:** 2016-08-23

**Authors:** Aung Pyae Phyo, Elizabeth A. Ashley, Tim J. C. Anderson, Verena I. Carrara, Charles J. Woodrow, Nicholas J. White, Francois Nosten

**Affiliations:** 1 Faculty of Tropical Medicine, Shoklo Malaria Research Unit, Mae Sot; 2 Faculty of Tropical Medicine, Mahidol-Oxford Tropical Medicine Research Unit, Mahidol University, Bangkok, Thailand; 3 Nuffield Department of Medicine, Centre for Tropical Medicine and Global Health, University of Oxford, United Kingdom; 4 Texas Biomedical Research Institute, San Antonio

To the Editor—We thank Professor Meshnick [1] and Dr Hastings and colleagues [2] for their interest in our publication [3]. Professor Meshnick believes that attempts to contain artemisinin resistance are futile because “all genetic evidence currently suggests that the K13 mutations pop up multiple times, independently, in different locations and are not spread spatially.” However, a very limited number of K13 alleles conferring artemisinin combination therapy (ACT) resistance are now replacing the throng of K13 alleles that appeared initially [4]. A more familiar pattern is emerging in which very few lineages of presumably fitter resistant parasites are increasing rapidly in frequency, causing rising rates of treatment failure and reinforcing the urgent need for an effective emergency containment response. The threat of spread from Asia to Africa should not be ignored: *dhfr* alleles resistant to pyrimethamine “popped up” several times in both Africa and Southeast Asia, but the highly resistant alleles that now predominate in Africa originated from Southeast Asia. Professor Meshnick says that “What the malaria control community needs is a ‘whack-a-mole’ approach, utilizing robust surveillance and targeted responses.” According to Wikipedia, the term “whack-a-mole” is used colloquially to denote a repetitious and futile task (https://en.wikipedia.org/wiki/Whac-A-Mole). We are concerned that this does indeed describe current containment approaches, which clearly have not worked. We believe that a balanced strategy involving containment and elimination in Southeast Asia combined with increased vigilance in Africa are required to manage resistance effectively.

Dr Hastings and colleagues draw attention to their modeling exercises, which claim to show that parasite clearance rates are insensitive and nonspecific measures of resistance. They questioned whether plans to contain artemisinin resistance should have been based on measures of parasite clearance viz “The use of infected red blood cell clearance rates as measures of drug effectiveness is particularly worrying” [5]. They also challenge the evidence that slow parasite clearance is heritable despite strong evidence from the largest and most detailed studies of parasite clearance and parasite genetics ever conducted that it is [6], and despite clear demonstration that a major gene (K13) underlies this trait [7–9]. We did not “point the journal readership to this work” [5] because we think the key biological assumptions they make on the mechanisms of parasite clearance are flawed (eg, note that most of the parasite clearance following artemisinin treatment in nonimmune populations is through pitting and *not* infected red cell clearance [10]), and we disagree with the conclusions of these 2 modeling papers for which there is no clinical evidence. Our study adds to mounting evidence that artemisinin resistance precedes partner drug resistance, and is therefore a harbinger of treatment failure—and that slow parasite clearance is the key clinical phenotype. On the question of dosage, many have pointed to the relatively low doses of artemether and dihydroartemisinin in current ACTs, but the dose of artesunate recommended and used in ACTs has always been 4 mg/kg. Although 2 mg/kg appeared to give maximum parasite clearance rates in a small pharmacodynamic study [11], the dose of artesunate we recommended and used initially in ACTs was 4 mg/kg precisely because of interindividual variability in pharmacodynamic responses [12]. On the other hand, despite disagreeing with most of their conclusions, we do strongly agree with Hastings et al that an informed, evidence-based debate is required to ensure that the threat of artemisinin resistance is properly addressed.
